# Male Alternative Reproductive Tactics and Associated Evolution of Anatomical Characteristics in Loliginid Squid

**DOI:** 10.3389/fphys.2019.01281

**Published:** 2019-10-15

**Authors:** José E. A. R. Marian, Lígia H. Apostólico, Chuan-Chin Chiao, Roger T. Hanlon, Noritaka Hirohashi, Yoko Iwata, Jennifer Mather, Noriyosi Sato, Paul W. Shaw

**Affiliations:** ^1^Department of Zoology, Institute of Biosciences, University of São Paulo, São Paulo, Brazil; ^2^Department of Life Sciences, National Tsing Hua University, Hsinchu, Taiwan; ^3^Marine Biological Laboratory, Woods Hole, MA, United States; ^4^Department of Life Sciences, Shimane University, Matsue, Japan; ^5^Atmosphere and Ocean Research Institute, University of Tokyo, Kashiwa, Japan, Japan; ^6^Department of Psychology, University of Lethbridge, Lethbridge, AB, Canada; ^7^Department of Fisheries, School of Marine Science and Technology, Tokai University, Shizuoka, Japan; ^8^Institute of Biological, Environmental and Rural Sciences, Aberystwyth University, Aberystwyth, United Kingdom; ^9^Department of Ichthyology & Fisheries Science, Rhodes University, Grahamstown, South Africa

**Keywords:** sexual selection, alternative phenotypes, ARTs, male dimorphism, consort, sneaker, Cephalopoda, Loliginidae

## Abstract

Loliginid squids provide a unique model system to explore male alternative reproductive tactics (ARTs) and their linkage to size, behavioral decision making, and possibly age. Large individuals fight one another and the winners form temporary consortships with females, while smaller individuals do not engage in male-male agonistic bouts but use various sneaker tactics to obtain matings, each with varying mating and fertilization success. There is substantial behavioral flexibility in most species, as smaller males can facultatively switch to the alternative consort behaviors as the behavioral context changes. These forms of ARTs can involve different: mating posture; site of spermatophore deposition; fertilization success; and sperm traits. Most of the traits of male dimorphism (both anatomical and behavioral) are consistent with traditional sexual selection theory, while others have unique features that may have evolved in response to the fertilization environment faced by each temporary or permanent male morph.

## Introduction

Since its formal conception nearly 150 years ago (Darwin, 1871), sexual selection has been an active field of evolutionary biology, particularly since the 1970’s [reviewed in Birkhead and Møller (1998), Birkhead and Pizzari (2002), Birkhead (2010), Parker and Pizzari (2015)]. We now understand that this powerful selective force operates through both intrasexual and intersexual mechanisms, as well as before and after mating. Pre-copulatory processes generally include male-male competition to access females, and female choice of males based on their assessment of male “quality.” Post-copulatory processes in polyandrous mating systems include sperm competition and cryptic female choice. Sperm competition, the contest between sperm from different males to access a female’s ova (Parker, 1970), leads to male adaptations (both anatomical and behavioral) that maximize fertilization success, including ejaculate traits (sperm placement, number, size, and performance). Cryptic female choice involves female control over male fertilization success (e.g., ejecting sperm or influencing their access to ova) (Eberhard, 1996).

Sexual selection drives the evolution of alternative reproductive tactics (ARTs). ARTs refer to discontinuous behavioral and other traits selected to maximize fitness in two or more alternative ways in the context of intraspecific and intrasexual reproductive competition (Oliveira et al., 2008). ARTs evolve when conspecific, intrasexual competitors find different solutions to reproductive competition. The concept of ARTs refers to alternative ways to obtain fertilizations (not just matings, since DNA studies in numerous taxa have shown that mating success does not predict fertilization success; e.g., Birkhead, 2010).

Males under intense sexual selection pressures may adopt one of two or more alternative patterns (Taborsky et al., 2008). If males physically compete for access to females and larger individuals win, small males may adopt an alternative way to achieve fertilizations through surreptitious mating (i.e., sneaker tactics). Male ARTs are found in numerous taxa and often involve a dominant tactic (guarder, territorial, bourgeois or consort) and an alternative way to obtain fertilizations (extra-pair, opportunistic, parasitic, sneaker or satellite) [reviewed in Oliveira et al. (2008)].

Sperm competition may be asymmetrical between males employing ARTs: dominant males gain priority access for their sperm, whereas extra-pair males may compensate by producing larger quantity or higher quality of sperm (Parker, 1990). In this context, the dominant male should invest in large body size and fighting behaviors, and the smaller in gonadal size and larger sperm size, speed or longevity (Taborsky, 1998). However, it is also important to consider spatial and temporal dynamics of fertilization when estimating the influence of sperm competition on the evolution of sperm traits. If the fertilization environment faced by each tactic is different, a dominant tactic may deviate from the classic role and produce larger, faster and more long-lasting sperm (Taborsky et al., 2018).

**FIGURE 1:**
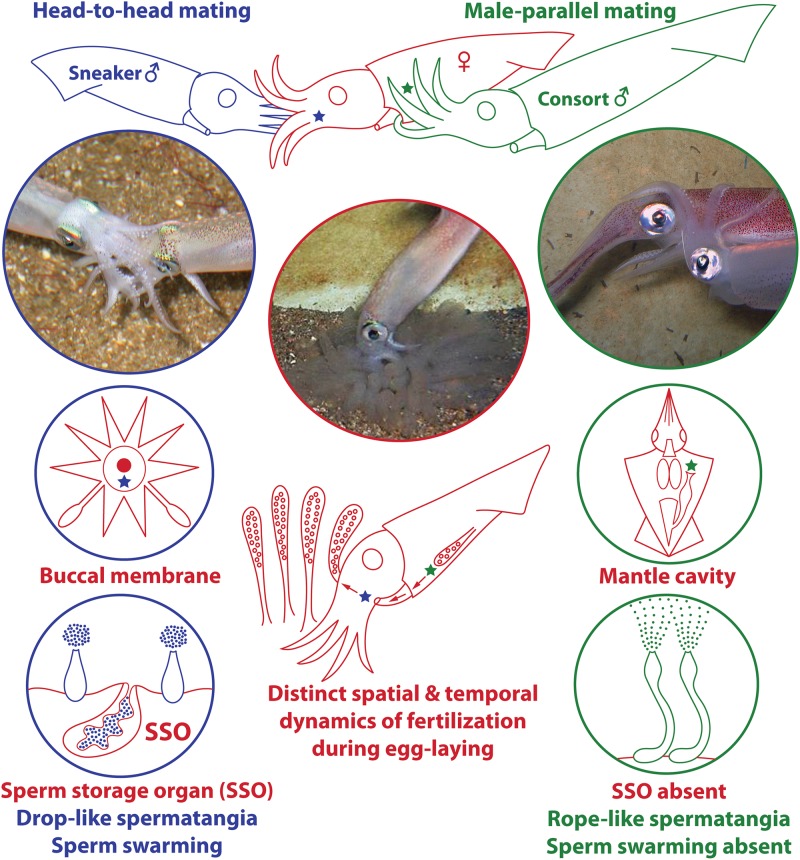
Summary of male ARTs and associated evolution of anatomical characteristics in loliginid squid. In several loliginids, large consort males (green) fight other males to gain access to females (red) and deposit spermatophores within the female mantle cavity near the oviduct opening (green star) through “male-parallel” mating. Small sneaker males (blue) usually employ furtive mating and deposit spermatophores near the female buccal seminal receptacle (blue star) through “head-to-head” mating; a sperm storage organ (SSO) is present at the female buccal membrane. Each site offers unique fertilization environments that differ, for example, in fertilization timing and success, which possibly led to the evolution of unique ejaculate traits by each male morph. The photographs and drawings used in this plate were adapted from figures originally published in Marian (2012b) and Apostólico and Marian (2018b); they are reproduced here with permission.

In this wider context of evolution of mating tactics, loliginid squid (Mollusca: Cephalopoda: Loliginidae) provide some unique behavioral and anatomical features with which to explore male sexual selection. Males of many species compete to guard and copulate with females. Unlike most animals, however, in some loliginid species there are two well-separated sites of spermatophore deposition and storage on the female body (Drew, 1911): on the buccal region near a sperm storage organ (seminal receptacle) or within the mantle cavity near the oviduct opening, associated with the two male mating tactics (Figure 1; “sneaker” vs. “consort,” respectively – Shashar and Hanlon, 2013; Iwata et al., 2015). In both cases, the spermatophores evert and attach themselves autonomously at the deposition site when transferred to the female, by means of a combination of mechanical and chemical processes – the sperm are then released from the distal tip of the attached “spermatangia” (i.e., everted spermatophores) and slowly disperse (Figure 1; Drew, 1919; Marian, 2012a, b, 2015). As fertilization occurs during egg-laying, deposition in the mantle cavity is likely a more successful tactic due to the proximity to the site of egg string extrusion (Figure 1; Iwata et al., 2005; Naud et al., 2016), but deposition in the buccal region from sneakers can also lead to fertilization as the egg string is held by the female in this region after extrusion but prior to placing it on the seabed (Figure 1; Buresch et al., 2009).

Much progress has been made in the last 25 years in understanding the squid mating system with the application of approaches including extensive *in situ* (e.g., Sauer et al., 1997; Hanlon et al., 2002, 2004; Shashar and Hanlon, 2013; Mather, 2016) and *ex situ* behavioral studies (e.g., Lin and Chiao, 2018), experimental manipulations of captive specimens (e.g., Iwata et al., 2005; Buresch et al., 2009; Saad et al., 2018), paternity analyses (from *ex situ* samples: Iwata et al., 2005; Buresch et al., 2009; from *in situ* samples: Shaw and Boyle, 1997; Shaw and Sauer, 2004; Naud et al., 2016), *in vitro* experimentation of the functioning of spermatophores (e.g., Iwata et al., 2015; Apostólico and Marian, 2017), spermatology (e.g., Iwata et al., 2011; Hirohashi and Iwata, 2013; Hirohashi et al., 2013, 2016a,b; Iida et al., 2017), gonadal/ejaculate expenditure (e.g., Iwata and Sakurai, 2007; Apostólico and Marian, 2018a; Iwata et al., 2018), and age and development (Apostólico and Marian, 2018b).

Here, we present a fresh perspective on male squid ARTs, and we reconstruct the evolution of reproductive characters and loliginid ARTs, discussing the interplay between sperm competition and fertilization environment in the evolution of ejaculate adaptations.

## Male Alternative Reproductive Tactics in Loliginids: Ca. 25 Years of Research

In the genus *Sepioteuthis*, which might be regarded as basal within the family, the behavioral and morphological specializations mentioned above are somewhat different. In *Sepioteuthis sepioidea*, both consort and sneaker tactics are present. Consort males will pair with a female for a day or so and mate in the “male-parallel” position and deposit spermatophores inside the mantle cavity (like *Loligo* and *Doryteuthis*), while small males (perhaps sneakers although they are seen paired with females for hours with no other male present) will pair briefly with females and will “slap” a spermatophore externally at or around the base of the arms. The female will grab the spermatophores and either spit them away (rejection) or place them near the seminal receptacle near the mouth (acceptance) (Moynihan and Rodaniche, 1982; Hanlon and Messenger, 2018). Interestingly, Mather (2016) reported that both sneakers and consorts mate similar to the second method explained above, and that consorts only mate in “male parallel” immediately before egg deposition. Mather (2016) also noted that the female may take spermatangia deposited on her dorsal arm bases into her mantle cavity, and that males shift from sneaker to consort tactics as they grow larger. *Sepioteuthis sepioidea* groups do not gather at spawning sites, and females reject consorts and sneakers often, even after a few successful spermatophore transfers (Mather, 2016). Males of this species conduct elaborate agonistic bouts and the winners pair with the female temporarily; presumed sneakers, however, do not engage in agonistic bouts (Moynihan and Rodaniche, 1982; Mather, 2016; Hanlon and Messenger, 2018). In *S. australis* observed at spawning sites (Jantzen and Havenhand, 2003), sneakers and consorts use postures and deposition sites that are basically similar to *S. sepioidea*, but female rejection of sneakers led to 67% of consorts and 33% of sneakers successfully transferring sperm. In captive *S. lessoniana* (Wada et al., 2005; Lin and Chiao, 2018) consort and sneaker males use different postures and different deposition sites, but smaller individuals who were rejected by females if they used the “consort” tactic could switch both posture and placement, demonstrating the facultative nature of switching tactics rapidly, depending on the behavioral context. *Sepioteuthis* spp. have complex mating systems that, unlike other loliginids, include distinctive courtship body patterns and behaviors by females and males (Hanlon and Messenger, 2018).

The tactics of male *Doryteuthis* and *Loligo* are somewhat different. Field and laboratory studies on three *Doryteuthis* species (*D. pealeii*, *D. pleii* and *D. opalescens*) and *Loligo reynaudii* during the 1990s and early 2000s revealed complex mating systems, which include communal spawning beds and at least two mating postures and sperm deposition sites, and a high degree of behavioral plasticity in both males and females (Hanlon, 1996, 1998; DiMarco and Hanlon, 1997; Hanlon et al., 1997, 2002, 2004; Sauer et al., 1997; Maxwell et al., 1998; Maxwell and Hanlon, 2000; Wada et al., 2005; Zeidberg, 2009). These studies provided substantial details of male ARTs of these species (Table 1). As in *Sepioteuthis*, the consort tactic consists of recurrent attempts to pair with females and repeated agonistic contests with other males. Consorts use a “male-parallel” mating posture and deposit their spermatophores within the female mantle cavity near the oviduct opening (Figure 1). Smaller sneaker males do not fight, but rather try quick extra-pair copulations in the “head-to-head” mating posture, placing their spermatophores near the female’s seminal receptacle located on the buccal membrane (Figure 1). *Doryteuthis pealeii* males have four mating tactics: consort, lone large male, surreptitious sneaker, and bold sneaker. The first two are large males, the last two are small males. None of these are separate genetic morphs, but rather facultative alternative behaviors depending on the size of the males and the combinations of large and small males that are present in different groupings within the mating arena (Shashar and Hanlon, 2013).

**TABLE 1:** Summary of male alternative reproductive tactics and related traits across Loliginidae.

						**Sperm swimming**
		**Mating**	**Sperm storage**	**Spermatangia**	**Sperm size**	**behavior**
**Species**	**ARTs**	**postures**	**sites**	**dimorphism**	**dimorphism**	**dimorphism**
*Doryteuthis pealeii*	SN/CO (1)	HH/MP (1)	BM/MC (2)	+(2, 3)^A^	?	?
*Doryteuthis pleii*	SN/CO (4)	HH/MP (4)	BM/MC (4)	+(4, 5)	+(4)	+(5)
*Doryteuthis opalescens*	SN/CO (6, 7)	HH/MP (6, 7)	BM/MC (7, 8)	?	?	?
*Heterololigo bleekeri*	SN/CO (9)	HH/MP (10)	BM/MC (10)	+(11)	+(11)	+(12)
*Loligo reynaudii*	SN/CO (13)	HH/MP (13)	BM/MC (14)	+(14)	?	+(15)
*Sepioteuthis australis*	SN/CO (16)	HH/MU/MP (16)	BM/HAR/MC (16)	?	?	?
*Sepioteuthis lessoniana*	SN/CO (17)	HH/MU/MP (18, 19)	BM/MC (17)	+(17)^B^	–(17)	?
*Sepioteuthis sepioidea*	SN/CO (20, 21)	HAR^C^/MP (20, 21)	BM/HAR/MC (20, 21)	?	?	?
*Uroteuthis edulis*	SN/CO (15)	?	BM/MC (15)	+(15)	+(15)	+(15)

*^*A*^Drew (1911: p. 359) illustration of the sagittal section of the female buccal membrane in *D. pealeii* is suggestive of the presence of “drop-like” spermatangia near the seminal receptacle, and Drew (1919: p. 424) spermatangium illustration clearly depicts a “rope-like” type; ^*B*^Lin et al. (2019) showed that sneaker and consort males of *S. lessoniana* exhibit different oral extremity of the cement body in spermatophores, suggestive of spermatangia dimorphism; ^*C*^Males and females of *S. sepioidea* rock together in parallel and then the male darts around and places spermatangia on her dorsal arm bases, from which she may take them to her buccal membrane (Hanlon and Messenger, 2018) or into the mantle cavity (Mather, 2016). References: (1) Shashar and Hanlon (2013); (2) Drew (1911); (3) Drew (1919); (4) Apostólico and Marian (2018a); (5) Apostólico and Marian (2017); (6) Hanlon et al. (2004); (7) Zeidberg (2009); (8) Fields (1965); (9) Iwata and Sakurai (2007); (10) Iwata et al. (2005); (11) Iwata et al. (2011); (12) Hirohashi et al. (2013); (13) Hanlon et al. (2002); (14) Iwata et al. (2018); (15) Hirohashi et al. (2016a); (16) Jantzen and Havenhand (2003); (17) Lin et al. (2019); (18) Wada et al. (2005); (19) Lin and Chiao (2018); (20) Mather, 2016; and (21) Hanlon and Messenger (2018). Abbreviations: ARTs, alternative reproductive tactics; BM, buccal membrane; CO, consort; HH, head-to-head; HAR, head/arm region; MC, mantle cavity; MP, male-parallel; MU, male-upturned; SN, sneaker; **+**, present; **−**, absent; ?, unknown.*

Courtship and postcopulatory mate guarding vary substantially in loliginids. Courtship is known only in *S. sepioidea*, and has been seen in both sneakers and consorts. There are only hints of courtship in males of *Loligo* or *Doryteuthis*, and it consists only of synchronized swimming next to the female; no specific body patterns have been reported. With the exception of *S. sepioidea*, sneakers neither court nor mate guard in loliginids (as known thus far). Mate guarding by consorts after copulation is common in the few species studied (Hanlon and Messenger, 2018), but it is generally temporary (but see Mather, 2016), and females can and do continue to fertilize and lay eggs without male accompaniment. The tactics decided by consort males at this stage of the mating system are unknown.

DNA fingerprinting has been used to study paternity (i.e., fertilization success of competing males) of wild or captive *D. pealeii*, *L. reynaudii*, *L. forbesii*, and *Heterololigo bleekeri*, confirming multiple paternity among the offspring in at least some broods in all species (Shaw and Boyle, 1997; Buresch et al., 2001, 2009; Shaw and Sauer, 2004; Iwata et al., 2005; Naud et al., 2016). Consorts achieve much higher reproductive success than sneakers in all cases, from ∼70% of overall fertilization in *L. reynaudii* (Naud et al., 2016) to ∼90% in *H. bleekeri* (Iwata et al., 2005). Moreover, the reproductive success of consort males is influenced by the interval between mating and egg-laying (Buresch et al., 2009). Also, non-random patterns of paternity within single egg strings may indicate cryptic female choice (Shaw and Sauer, 2004; Naud et al., 2016).

Male ARTs always involve behavior, but also generally include distinct sets of morphological and physiological attributes (Taborsky et al., 2008). Accordingly, male dimorphism is present in the four loliginids (*H. bleekeri*, *D. pleii*, *L. reynaudii*, and *Uroteuthis edulis*) studied in detail to date, expressed as dimorphic ejaculates (Iwata and Sakurai, 2007; Iwata et al., 2015, 2018; Apostólico and Marian, 2017, 2018a,b; Table 1). Large and small males generally follow consort and sneaker tactics, with sneaker males developing small “drop-like” spermatangia, while consort males produce larger and elongate “rope-like” spermatangia (Figure 1; Iwata and Sakurai, 2007; Iwata et al., 2015, 2018; Apostólico and Marian, 2017, 2018a,b; Table 1). This tactic-associated dimorphism even extends to differences in sperm size, with sneakers producing consistently larger sperm (Iwata et al., 2011; Table 1).

Besides differences in spermatophore and sperm size (Table 1), some of the most intriguing adaptations of each ejaculate type are the duration of sperm release from the spermatangium, being much longer (ca. 5 h) in sneaker than consort spermatangia (ca. 2 h; Apostólico and Marian, 2017), and sperm swimming behavior after release, showing an aggregative behavior – swarming – in sneaker sperm (Figure 1; Iwata et al., 2011; Hirohashi and Iwata, 2013; Hirohashi et al., 2013, 2016a; Apostólico and Marian, 2017; Table 1). Physiological investigations of *H. bleekeri* demonstrated that swarming occurs because sneaker sperm migrate toward acidic environments (pH-taxis; Hirohashi et al., 2013).

Although there are mating postures and behaviors typical of each male tactic, with head-to-head (or male-upturned) mating typical for sneakers and male-parallel mating and agonistic behavior typical for consorts, they are not always exclusive. Mating behavior may vary according to the behavioral context in the mating arena. For example, consort males may copulate in the head-to-head position if the female is far from spawning (*H. bleekeri*; Iwata et al., 2005; *S. lessoniana*; Wada et al., 2005). In *D. pealeii*, sneakers can immediately switch their behavior when a consort male is removed and perform male-parallel mating, and then just as rapidly switch back to sneaker tactics and head-to-head mating when a consort male appears and pairs temporarily with the female (Hanlon et al., 1997; Shashar and Hanlon, 2013). Female choice (*S. lessoniana*; Lin and Chiao, 2018) or relative size of males (*S. lessoniana*; Wada et al., 2005) can also influence mating postures and behaviors.

In other animal taxa, ARTs can be either fixed or plastic, and in the latter case either simultaneous or sequential (Taborsky et al., 2008). In *H. bleekeri* ARTs are apparently fixed, with spermatophore length discontinuous across body size suggesting two distinct morphs (Iwata and Sakurai, 2007). However, in some loliginids the expression of ARTs can be sequential (e.g., *S. sepioidea*), and this can be observed only through long-term behavioral observations (Moynihan and Rodaniche, 1982; Mather, 2016). Males of *D. pleii* of intermediate size and age show a transition of sneaker to consort-like ejaculates inside the reproductive tract (Apostólico and Marian, 2018b), as well as a transition in mating behavior (Apostólico and Marian, 2019), so male dimorphism may be sequential in this species also (but see alternative hypotheses in Apostólico and Marian, 2018b). As intermediate-sized males of *L. reynaudii* (Iwata et al., 2018), *D. pealeii* (Shashar and Hanlon, 2013), and *S. lessoniana* (Lin et al., 2019) may display flexible tactics, their male ARTs could be sequential, too, but further studies are required.

## Dimorphic Male Adaptations: The Interplay Between Sperm Competition and Fertilization Environment

Fertilization in loliginids generally occurs during egg-laying, first near the oviduct opening within the mantle, and then on the buccal membrane when the egg string is held within the female’s arms (Figure 1; e.g., Drew, 1911; Sauer et al., 1997; Hanlon et al., 2002; Buresch et al., 2009; Iwata et al., 2015; Naud et al., 2016). This “confined external fertilization,” occurring potentially in two different sites, creates a useful model to investigate the interplay between the pressures of sperm competition and fertilization environment. Some male adaptations follow the predictions of sperm competition theory, which predicts that if sneakers face a behavioral disadvantage they should show higher gonadal investment (Parker, 1990). In *D. pleii* sneakers have higher gonadal expenditure than consorts and the latter invest more in somatic growth (Apostólico and Marian, 2018a). Other adaptations are influenced by the respective sperm storage sites, which comprise a unique fertilization environment, differing in:

(1) Sources of spermatozoa: in the mantle cavity the only source is the attached spermatangia, but the buccal membrane has two distinct sources, the attached spermatangia and the seminal receptacle (Figure 1). Attached spermatangia of the mantle cavity and the buccal membrane provide sperm from recent matings, but the seminal receptacle possibly stores sperm from much earlier mating events (e.g., Hanlon, 1996; Hanlon et al., 2002);
(2) Fertilization success: due to proximity to the oviduct opening, fertilization success is higher for deposition in the mantle cavity site (Figure 1; e.g., Iwata et al., 2005; Naud et al., 2016);
(3) Interval between mating and fertilization: it can be longer for the buccal membrane as it has a sperm storage organ that may store sperm for a considerable period of time (Figure 1; e.g., Hanlon, 1996; Hanlon et al., 2002). Head-to-head mating may occur hours before spawning, while male-parallel usually occurs near or during spawning (Iwata et al., 2005; Wada et al., 2005);
(4) Physical features of each site (see Iwata et al., 2011): there may be differences in the risk of sperm dilution (e.g., while the buccal membrane is a more external site, the mantle cavity may exhibit considerable turbulence due to constant mantle contractions), as well as differences in pH, viscosity and salinity. Because fertilization occurs during egg-laying, there may be changes in viscosity of the jelly matrix or expansion (e.g., swelling) of the egg string as it is extruded from the mantle cavity (Boletzky, 1986) then exposed to seawater as it is moved to the buccal membrane.

In this context, differences in sperm size and spermatangia shape and function (Figure 1) are possibly associated with physical constraints specific to each deposition site (Iwata et al., 2011; Apostólico and Marian, 2017). Also, the slower sperm release (Apostólico and Marian, 2017) and longer sperm viability (Hirohashi et al., 2016b) in sneakers may be associated with the longer interval between mating and fertilization for spermatangia attached on the buccal membrane. Additionally, the smaller sneaker spermatangium is filled with fewer sperm (Iwata and Sakurai, 2007; Iwata et al., 2011; Apostólico and Marian, 2018a): if many oocytes are fertilized by sperm in the mantle cavity before reaching the buccal membrane, then sneakers may use fewer sperm per mating and invest in more mating events (Apostólico and Marian, 2018a).

Sperm size evolution has been a central issue in postcopulatory sexual selection theory. If a longer flagellum results in higher swimming speed, a classic prediction is that intense sperm competition could lead to larger sperm sizes (e.g., Snook, 2005). Also, due to the asymmetry in sperm competition (Parker, 1990), sneakers may expend more in gametic traits (e.g., sperm size). Interestingly, sneaker spermatozoa of *H. bleekeri* and *D. pleii* are 50% (Iwata et al., 2011) and 15% (Apostólico and Marian, 2018a) longer than consort sperm, respectively. However, at least for *H. bleekeri*, this difference in size does not result in higher swimming speed and is not related to competition for space within the seminal receptacle (Iwata et al., 2011). Therefore, sperm competition alone does not explain sperm dimorphism in this species (Iwata et al., 2011).

**FIGURE 2:**
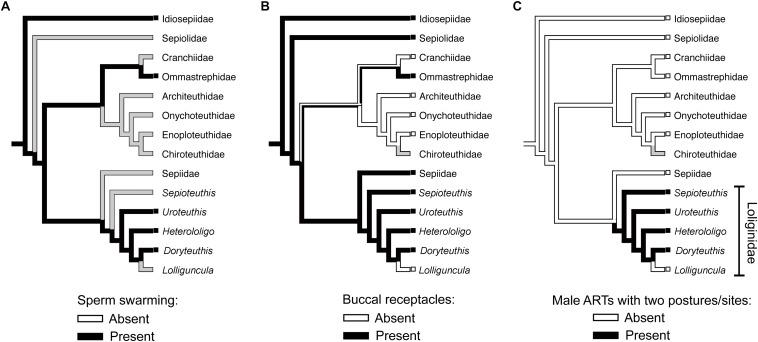
Parsimonious ancestral state reconstructions (ASR) of reproductive characters based on the topology for Decapodiformes of Lindgren and Anderson (2018). Character states are traced and indicated by colored branches (i.e., ancestral reconstruction) and colored boxes to the left of taxon names (i.e., the observed character state in the respective taxon). White and black colors indicate absence and presence, respectively. Presence of more than one color in branches indicates equivocal (i.e., uncertain) reconstruction, and gray color indicates equivocal reconstruction owing to missing data. **(A)** ASR of sperm swarming. **(B)** ASR of buccal seminal receptacles. **(C)** ASR of alternative reproductive tactics involving two mating postures, two sperm deposition sites (buccal membrane and mantle cavity) and ejaculate dimorphism. The methodology used in the analyses is detailed in the Supplementary Material.

Differences in sperm behavior could be associated with the spermatophore deposition site. The self-swarming trait of sneaker sperm (Figure 1) could be linked to collective sperm migration toward the seminal receptacle on the buccal membrane (Hirohashi and Iwata, 2013), and to slowing down sperm release from the spermatangium (i.e., by retaining sperm near its tip; Apostólico and Marian, 2017). Hirohashi et al. (2016a) hypothesized that sperm swarming was a primitive ejaculate attribute conserved in sneakers but lost in consort males who use mantle cavity deposition. We investigated this hypothesis with the available literature, using parsimonious ancestral state reconstructions based on a recent phylogenetic hypothesis for Decapodiformes, a large clade including squids, cuttlefishes, bobtail squids and ram’s horn squid (Figure 2). The analyses indicate that sperm swarming and buccal receptacles were present in the decapodiform ancestor, but that ARTs typical of some loliginids (i.e., two mating postures, two sperm deposition sites and ejaculate dimorphism) evolved later (Figure 2), tending to support (Hirohashi et al., 2016a).

If sperm swarming and buccal seminal receptacles, but not male ARTs, are plesiomorphic within Loliginidae, swarming would not be an adaptation of loliginid sneaker males but could be related to sperm storage in buccal receptacles (Hirohashi and Iwata, 2013). In contrast, consort sperm that diffuse after release from the spermatangium (Figure 1) presumably evolved associated with the changes in male ARTs within Loliginidae. The loss of swarming in consort sperm, maybe through the evolution of shorter sperm tails (see Iida et al., 2017), could be the result of relaxed selection due to the absence of a sperm storage organ in the mantle cavity, or increased selection due to sperm competition. Parallel mating is usually performed near or during spawning (Iwata et al., 2005; Wada et al., 2005; Buresch et al., 2009), and consorts are frequently replaced in some species (Hanlon et al., 2002; Shashar and Hanlon, 2013), so intense sperm release should guarantee a higher number of fertilizations for the consort male as more oocytes leaving the oviduct would be fertilized. If sperm swarming could delay sperm release from the sneaker spermatangium (Apostólico and Marian, 2017), then the loss of swarming in consorts is likely an adaptation to sperm competition when the physical constraints of external fertilization are relaxed.

## Future Directions

As described herein, loliginid squids provide a unique model group to study sexual selection due to the presence of two sperm deposition sites within the female body, each offering distinct fertilization environments for male gametes (Figure 1). However, some basic mechanisms operating in the squid mating system are still obscure, hindering its wide adoption as a model system to test sexual selection theory. Although all research teams involved with the present paper will continue in their respective areas to fill major gaps in our knowledge, we urge interdisciplinary approaches. A combination of tests of behavioral, genetic, reproductive biology and functional morphology data are needed to unravel the complexities of the loliginid squid mating system. We need:

(1) *In situ* behavioral studies (e.g., Shashar and Hanlon, 2013; Mather, 2016; Naud et al., 2016): because they reveal the full range of male ARTs and female choice under natural conditions that cannot all be duplicated in lab studies. Long-term field observations could clarify changes in ARTs across ontogeny since small (younger) squids tend to be sneakers and larger (older) squids consorts.
(2) Female roles: male tactics and dimorphism are likely to be affected by female choice. Apart from *S. sepioidea*, in which the females actively manipulate spermatangia received during mating (Moynihan and Rodaniche, 1982; Mather, 2016; Hanlon and Messenger, 2018), we do not know all of the tactics that females use to exert choice. Manipulation of egg-string extrusion and position within the arms and sperm release from the seminal receptacle could bias fertilization success toward particular males (Naud et al., 2016), and pumping the mantle cavity after male-parallel mating could eject consort spermatangia (Buresch et al., 2009).
(3) Spawning context: some loliginids form dense spawning aggregations and large open spawning beds, providing opportunities both for male and female promiscuity with both pre- and post-copulatory sexual selection (Hanlon and Messenger, 2018). Others (e.g., *S. sepioidea* and *H. bleekeri*) do not form large aggregations and use hard substrates for egg attachment (Jereb and Roper, 2010; Mather, 2016; Hanlon and Messenger, 2018). Do these differences in mating/spawning conditions affect the male ARTs?
(4) Fertilization dynamics: how exactly are loliginid eggs fertilized in each site? The egg string is initially formed within the mantle cavity (Boletzky, 1986), but is sperm penetration more difficult by the time the string reaches the buccal membrane, where the string is fully expanded? Sperm stored in the seminal receptacle or from spermatangia recently placed there by sneaker males would only have access to fertilization at this time. Knowledge of the structure and formation of the egg case (Iwata et al., 2019) is necessary to understand this.
(5) Sperm competition: we lack understanding of inter- and intra-tactic sperm competition, the interplay between it and the fertilization environment, and how they have influenced adaptations at both individual and gametic levels. The role of mate guarding in sperm competition is unknown but of probable importance to greater fertilization success by the guarding male. The extent of sperm swarming throughout the Cephalopoda is largely unknown (see Figure 2A and Table 1) and requires investigation.
(6) Behavioral plasticity: How diverse is this plasticity with respect to consort vs. multiple forms of sneaking in different species? Does plasticity in mating behaviors affect male dimorphism, especially in structural and physiological differences in ejaculates?
(7) Expression of ARTs: how do genetic and environmental factors underlie the expression of male ARTs, especially in the physiological transition between dimorphic males?
(8) Evolution of male ARTs: there are ten genera in the Loliginidae, but at present we have information about either behavior or physiology for only four genera, and not the whole spectrum of information for any species. Cross-disciplinary and comparative studies are needed across the range of loliginid species.

Nevertheless, a substantial increase in knowledge of loliginid male ARTs has been achieved within a relatively short period (1996–2019), and we look forward to the next 25 years of research on this interesting model system.

## Data Availability Statement

All datasets generated for this study are included in the manuscript/Supplementary Files.

## Ethics Statement

Ethical review and approval was not required for the animal study because all data was compiled from the literature. No animals were collected or used in experiments.

## Author Contributions

All authors contributed intellectually to the work, provided data and words to the manuscript versions, and edited the manuscript and approved it for publication.

## Conflict of Interest

The authors declare that the research was conducted in the absence of any commercial or financial relationships that could be construed as a potential conflict of interest. The handling Editor declared a shared committee membership, though no other collaboration, with one of the authors, JM, in the Cephalopod International Advisory Council.
