# Supplementary material for: Male Alternative Reproductive Tactics and Associated Evolution of Anatomical Characteristics in Loliginid Squid
Source: Front Physiol. 2019 Oct 15;10:1281. doi: 10.3389/fphys.2019.01281 (PMC6803530; doi:10.3389/fphys.2019.01281)
Supplement: Supplementary file 1 [file Data_Sheet_1.pdf]

Supplementary Material

1. Methodology

The distribution of reproductive characters' states was analyzed based on a recent phylogenetic tree for Decapodiformes (Lindgren and Anderson, 2018) using Mesquite 3.6 (Maddison and Maddison, 2018). A species-character matrix (14 terminal taxa, 3 unordered characters; see below) was built based on literature information. Parsimonious character optimizations were performed using the "Trace character history" function of Mesquite 3.6. To facilitate the discussion for a wider audience, species names of the original tree were replaced by higher taxonomic ranks (family or, in the case of loliginids, genus names).

1.1. Character and character states description

I. Sperm swarming – presence: (0) absent; (1) present. Sperm swarming (i.e., the ability of sperm to aggregate after release from the spermatangium) was confirmed to be present in *Heteroligo bleekeri*, *Uroteuthis edulis*, *Doryteuthis pleii*, *Idiosepius paradoxus* (Idiosepiidae) and *Todarodes pacificus* (Ommastrephidae) (Hirohashi et al., 2016; Apostólico & Marian, 2017). Therefore, we have considered sperm swarming to be present in their respective lineages. Although swarming is absent in *Euprymna morsei* (Sepiolidae; Hirohashi et al., 2016), we have considered the state as "uncertain" for Sepiolidae, because this large clade includes some species with a seminal receptacle on the ventral buccal membrane (e.g., Sepiadariidae; Nesis, 1995), and we lack information on swarming for them.

II. Buccal seminal receptacles: (0) absent; (1) present. Seminal receptacles are sperm storage organs that receive sperm released from spermatangia attached on the buccal membrane. Ommastrephid squids have numerous seminal receptacles around the buccal membrane; idiosepiids, sepiids, loliginids and some sepiolids have only one main organ located ventrally on the buccal membrane (e.g., Marian, 2015). Coding for this character was based on Nesis (1995), Marian (2015) and Hanlon and Messenger (2018). For some species, there is sufficient knowledge to confirm the absence of buccal receptacles in females (e.g., Architeuthidae, Onychoteuthidae), but for poorly studied species (e.g., Chiroteuthidae) we have preferred to code the state as "uncertain".

III. Alternative reproductive tactics involving two mating postures, two sperm deposition sites (buccal membrane vs. mantle cavity) in the female, and ejaculate dimorphism: (0) absent; (1) present. ARTs in some loliginids (sneaker vs. consort tactics) may include each a distinct set of behavioral and ejaculate traits, although in some species there is some behavioral flexibility within each tactic. For the state "present", we have only considered species for which two mating postures and two sperm deposition sites have been recorded along with ejaculate dimorphism (distinct spermatangium and sperm types): *Heterololigo bleekeri* (Iwata and Sakurai, 2007; Hirohashi and Iwata, 2013); *Doryteuthis pleii* (Apostólico and Marian, 2017, 2018a, 2018b); *Loligo reynaudii* (Hirohashi et al., 2016; Iwata et al., 2018); and *Uroteuthis edulis* (Hirohashi et al., 2016). For *Sepioteuthis*, there is evidence of two mating postures and two deposition sites (e.g., Mather, 2016; Hanlon & Messenger, 2018; Lin & Chiao, 2018), but still not for ejaculate dimorphism. However, sneaker and consort males of *S. lessoniana* show different oral extremity of the cement body in spermatophores (Lin et al., 2019), suggesting at least spermatangia dimorphism. Thus, we arguably coded the state as "present". For Chiroteuthidae, due to the scarce information for this taxon, we coded the state as "uncertain". For the remaining terminals, there is sufficient information to affirm that two mating postures and the two simultaneous sperm deposition sites are absent (Nesis, 1995; Marian, 2015; Hanlon and Messenger, 2018).

1.2. Taxa-character matrix

	CHARACTERS		
TAXA:	I	II	III
Idiosepiidae	1	1	0
Sepiolidae	?	1	0
Cranchiidae	?	0	0
Ommastrephidae	1	1	0
Architeuthidae	?	0	0
Onychoteuthidae	?	0	0
Enoploteuthidae	?	0	0
Chiroteuthidae	?	?	?
Sepiidae	?	1	0
Sepioteuthis	?	1	1
Uroteuthis	1	1	1
Heterololigo	1	1	1
Doryteuthis	1	1	1
Lolliguncula	?	0	0

1.3. References

Apostólico, L. H., and Marian, J. E. A. R. (2017). Dimorphic ejaculates and sperm release strategies associated with alternative mating behaviours in the squid. *Journal of Morphology* 278, 1490-1505.

Apostólico, L. H., and Marian, J. E. A. R. (2018a). Dimorphic male squid show differential gonadal and ejaculate expenditure. *Hydrobiologia* 808, 5-22.

Apostólico, L. H., and Marian, J. E. A. R. (2018b). From sneaky to bully: Reappraisal of male squid dimorphism indicates ontogenetic mating tactics and striking ejaculate transition. *Biological Journal of the Linnean Society* 123, 603-614.

Hanlon, R. T., and Messenger, J. B. (2018). *Cephalopod Behaviour*, second ed. Cambridge University Press, Cambridge.

Hirohashi, N., and Iwata, Y. (2013). The different types of sperm morphology and behavior within a single species: Why do sperm of squid sneaker males form a cluster? *Comunicative and Integrative Biology* 6, e26729.

Hirohashi, N., Iida, T., Sato, N., Warwick, S. H., and Iwata, Y. (2016). Complex adaptive traits between mating behaviour and post-copulatory sperm behaviour in squids. Reviews in Fish Biology and Fisheries 26(3), 601-607.

Iwata, Y., and Sakurai, Y. (2007). Threshold dimorphism in ejaculate characteristics in the squid *Loligo bleekeri. Marine Ecology Progress Series* 345, 141-146.

Iwata, Y., Sauer, W. H. H., Sato, N., and Shaw, P. W. (2018). Spermatophore dimorphism in the chokka squid *Loligo reynaudii* associated with alternative mating tactics. *Journal of Molluscan Studies* 84, 157-162.

Lin, C. Y., and Chiao, C. C. (2018). Female choice leads to a switch in oval squid male mating tactics. *Biological Bulletin* 233, 219-226.

Lin, C. Y., Chen, C. S., and Chiao, C. C. (2019). The overlapping reproductive traits of the two male mating types of the oval squid *Sepioteuthis lessoniana*. *Fisheries Science* 85, 339-347.

Lindgren, A. R., and Anderson, F. E. (2018). Assessing the utility of transcriptome data for inferring phylogenetic relationships among coleoid cephalopods. Molecular Phylogenetics and Evolution 118, 330-342.

Maddison, W. P., and Maddison, D. R. (2018). Mesquite: a modular system for evolutionary analysis. Version 3.6. https://www. mesquiteproject. org.

Marian, J. E. A. R. (2015). Evolution of spermatophore transfer mechanisms in cephalopods. *Journal of Natural History* 49, 1423-1455.

Mather, J. (2016). Mating games squid play: reproductive behaviour and sexual skin displays in Caribbean reef squid *Sepioteuthis sepioidea*. *Marine and Freshwater Behaviour and Physiology* 49(6), 359-373.

Nesis, K. N., 1995. Mating, spawning, and death in oceanic cephalopods: a review. *Ruthenica* 6, 23–64.
